# AMBULATORY INDIVIDUALS WITH SPINAL CORD INJURY USING WALKING AIDS GENERALLY DO NOT REPORT BETTER LONG-TERM OUTCOMES THAN WHEELCHAIR USERS: RESULTS FROM THE INSCI-NL SURVEY

**DOI:** 10.2340/jrm.v58.44939

**Published:** 2026-06-30

**Authors:** Marthe C. G. LANGERWERF, Karin POSTMA, Janneke STOLWIJK-SWUSTE, Marga TEPPER, Majanka H. HEIJENBROK-KAL, Marcel W. M. POST, Rita J. G. VAN DEN BERG-EMONS, Rutger OSTERTHUN

**Affiliations:** 1 Department of Rehabilitation Medicine, Erasmus MC, University Medical Centre Rotterdam, Rotterdam, the Netherlands; 2 Rijndam Rehabilitation Centre, Rotterdam, the Netherlands; 3 Knowledge Centre Rehabilitation Medicine, UMC Utrecht, the Netherlands; 4 De Hoogstraat Rehabilitation, Utrecht, the Netherlands

**Keywords:** dependent ambulation, health status, International Classification of Functioning, mobility limitation, quality of life, spinal cord injuries

## Abstract

**Objective:**

Greater mobility independence after spinal cord injury is thought to lead to better long-term outcomes. However, evidence is inconclusive for ambulation compared with wheelchair dependency. This study aimed to describe and compare long-term outcomes across mobility levels in individuals with spinal cord injury.

**Design:**

Cross-sectional survey.

**Subjects:**

Community-dwelling individuals with spinal cord injury in the Netherlands.

**Methods:**

Data were collected via the Dutch arm of the International Spinal Cord Injury (InSCI-NL) Survey (2018). Mobility level was assessed using the modified Spinal Cord Independence Measure-Self-Report. Long-term outcomes were compared across mobility levels, using generalised linear models.

**Results:**

253 participants were included with a mean (standard deviation) age of 58.7 (13.6) years and median [interquartile range] time since injury of 10 [5.0–22.0] years. Electric wheelchair users reported lower functional independence, and more participation problems and secondary health conditions compared with manual wheelchair users (*p* < 0.001). Ambulatory individuals using walking aids reported no better outcomes than manual wheelchair users, except for functional independence (*p* < 0.001). Ambulatory individuals without walking aids reported more paid work than those with walking aids (62.7% vs 34.3%, *p* < 0.001).

**Conclusion:**

In general, electric wheelchair users reported the least favourable long-term outcomes. Remarkably, ambulatory individuals using walking aids generally did not report better outcomes than manual wheelchair users.

## LAY ABSTRACT

A spinal cord injury can greatly affect a person’s mobility and independence. Although walking is often seen as a sign of better recovery, it may not necessarily lead to better long-term outcomes. In this study, we described and compared long-term outcomes in community-living individuals with spinal cord injury in the Netherlands across mobility levels. Our findings show that individuals using electric wheelchairs generally experience greater challenges and lower functional independence than those using manual wheelchairs. Importantly, individuals who walk with aids generally did not report better outcomes than manual wheelchair users. These results suggest that walking ability alone does not guarantee better long-term outcomes. This insight increases awareness of the challenges of ambulatory individuals with spinal cord injury, supporting healthcare professionals in improving care to meet their needs.

A spinal cord injury (SCI) has a profound impact on an individual’s long-term outcomes, including functioning, health, and well-being (1). Recovery of functional independence after SCI varies widely and is largely determined by injury characteristics (2). A key, and most visible, part of functional independence is the ability to move around, i.e., mobility level. Mobility levels vary, in increasing order of independence, from dependence on an electric or manual wheelchair to being able to ambulate with or without walking aids (3). Individuals with more independent mobility levels are generally considered less affected by the SCI and are expected to achieve better long-term outcomes.

Several studies comparing electric with manual wheelchair users support this assumption, demonstrating more favourable outcomes in individuals with a more independent mobility level (4, 5). However, this is not clear for manual wheelchair users compared with ambulatory individuals, although ambulation is a high priority among individuals with SCI (6). Indeed, studies show several physical and psychosocial benefits of ambulation and locomotor training (7–9). On the other hand, it is increasingly emerging that ambulatory individuals with SCI experience fatigue, stigma, emotional distress, isolation, and several barriers to participation in society (10–13). A study comparing wheelchair users with ambulatory individuals found that ambulatory individuals reported substantial problems in activities and participation, and their quality of life and mental health were similar to those of wheelchair users (5). In contrast, another study reported that ambulation, compared with wheelchair use, was associated with better participation (14). However, this study reported a mixed pattern of favourable and unfavourable outcomes on health and well-being, and reported that those who depend on others for assistance with ambulation reported poorer outcomes than wheelchair users (14).

Concerning long-term outcomes in ambulatory individuals, it is important to note that there is a considerable variation in how the individuals move around in their daily lives. Ambulation ranges from individuals with limited walking ability who use walking aids to those who walk without aids and are even unlimited in their walking distance. This variation in the level of ambulation may impact long-term outcomes. For example, although walking aids may be necessary to improve balance and mobility, several disadvantages have been reported (15, 16). These disadvantages include interference with balance and excessive strain leading to pain and fatigue, factors known to impact well-being (15, 16). Furthermore, individuals with limited walking ability may be more dependent on assistance from others for ambulation, which is associated with less favourable long-term outcomes (14).

Understanding long-term outcomes in individuals with different mobility levels following SCI is essential to improve treatment strategies and outcomes for these subgroups. Recent findings and clinical experience suggest that ambulatory individuals may have less favourable long-term outcomes than previously assumed, highlighting the need for a deeper understanding of this population. This topic is increasingly important because of a growing number of individuals with SCI who can achieve a more independent mobility level, due to an increasing incidence of incomplete SCI and advancements in the treatment of SCI (17, 18). Therefore, this study aimed to describe the long- term outcomes related to functioning, health, and well-being among subgroups of community-living individuals with different mobility levels after SCI in the Netherlands. Second, we aimed to compare long-term outcomes across the subgroups, with a particular focus on comparing the adjacent groups: (*i*) electric wheelchair and manual wheelchair users, (*ii*) manual wheelchair users and ambulatory individuals using walking aids, and (*iii*) ambulatory individuals with and without walking aids. We hypothesized that individuals with more independent mobility levels would consistently report more favourable long-term outcomes in functioning and health compared with those with more dependent mobility levels.

## METHODS

Data were collected with the Dutch arm of the International SCI (InSCI) community survey (InSCI-NL) between January and October 2018. The InSCI survey was conducted in 22 countries to study the lived experience of individuals with SCI (19, 20). This comprehensive survey addressed the domains of the International Classification of Functioning, Disability, and Health (ICF) – Core Set for SCI in the long-term context (21). A more detailed description can be found in the study protocol of the overarching InSCI survey (25). A Dutch report on the preliminary results of long-term outcomes based on this InSCI-NL survey has been published previously (22). The InSCI survey adhered to ethical standards, including the Declaration of Helsinki. For InSCI-NL, the Dutch Medical Research Ethics Committee of the University Medical Centre Utrecht reviewed the study protocol and provided a waiver for formal ethical approval under the Dutch law for regulation of medical research in human beings (Reference number 17/539).

### Participants

The InSCI-NL survey was conducted among adults diagnosed with a traumatic or non-traumatic SCI, who were living in the community and had previously received inpatient treatment, outpatient treatment, or outpatient consultations in one of three rehabilitation centres in the Netherlands with specialised SCI units: Rijndam Rehabilitation in Rotterdam, De Hoogstraat Rehabilitation in Utrecht, and the Centre of Rehabilitation of the University Medical Centre Groningen in Groningen. These rehabilitation centres are located in the western, middle, and northern regions of the Netherlands, covering urban and rural areas. The inclusion criteria were resident of the Netherlands, 18 years or older, and having sufficient command of the Dutch language to complete the survey. Exclusion criteria were current admission for inpatient rehabilitation, or an SCI resulting from congenital abnormality or neurodegenerative process. Additionally, for this study, participants with missing information on mobility level were also excluded.

### Procedure

A random sample of 847 individuals was drawn from databases of the three participating rehabilitation centres and invited to participate by postal mail. They were provided a link to a secure local website to complete the survey online. Alternatively, participants could complete a paper version of the questionnaire and return it by postal mail. Non-responders received one reminder after a few weeks. By completing the survey, participants gave their consent to participate in the study.

### Measures

The InSCI-NL survey consisted of a Dutch translation of the international survey (20, 23), which is based on the ICF core sets of SCI and Rehabilitation (21). In addition, the Dutch survey was supplemented with questions regarding the level of physical activity (PA).

### Demographic and injury characteristics

The survey began with questions on demographic and injury characteristics. Demographic characteristics included current age, sex (male/female), marital status, and education level. Participants reported their marital status by selecting one of the following options: single, married, cohabiting, or in a partnership; separated or divorced; and widowed. These responses were dichotomised into “married, cohabiting or in a partnership” (yes/no). Education was reported using the following categories: no schooling or never completed, primary, lower secondary, higher secondary, post-secondary, short tertiary, bachelor or equivalent, and master or equivalent. These were subsequently dichotomised into having completed higher education (yes/no), defined as completion of tertiary education (i.e., short tertiary or higher). Injury characteristics included cause of injury, injury level (tetraplegia/paraplegia), completeness of injury (complete/incomplete), and time since injury (TSI) (19, 20).

### Mobility level

Mobility level was assessed using the mobility subscale of the modified Spinal Cord Independence Measure-Self Report (m-SCIM-SR) (24). Based on the item from the m-SCIM-SR on the mode of moving over a distance of 10–100 m, mobility levels were divided into four subgroups; (*i*) electric wheelchair, including those who needed total assistance, electric wheelchair, or partial assistance to operate a manual wheelchair, (*ii*) manual wheelchair, including those who were independent in a manual wheelchair or needed supervision while walking, (*iii*) walking with aid, including those who walked with a walking frame or crutches swinging forward with both feet at a time, walked with crutches or two canes setting one foot before the other, walked with one cane, or walked with a leg orthosis only, and (*iv*) walking without aids, including those who walked without aid (24). Individuals who only walked under supervision were classified as manual wheelchair users, as their primary mode of mobility was expected to be wheelchair driving, with walking limited to short distances under the guidance of family members or a therapist.

### Long-term outcomes

*Functional independence* was assessed using the *self-care* and the *bladder and bowel management* subscales of the m-SCIM-SR. The subscale for self-care consists of six items, and the subscale for bladder and bowel management consists of seven items (24). Response options ranged from 0 to 5, with scores adjusted so that a higher score indicated greater independence. Per subscale, a sum score was calculated ranging from 0 to 20 for self-care and from 0 to 22 for bladder and bowel management.

The participants were asked whether they had *paid work* (yes/no). Analyses concerning paid work were conducted on participants younger than 66, reflecting the statutory retirement age in the Netherlands at the time of survey (19).

*Participation problems* in social activities were assessed using items from the Model Disability Survey (MDS) (19). On a 5-point scale, participants were asked to rate eight questions regarding participation problems over the past 4 weeks, with 1 “no problem” and 5 “very serious problem”. A sum score was calculated, ranging from 8 to 40, with higher scores indicating more or more severe participation problems (19).

*PA* in the past 7 days was assessed using two items on moderate and vigorous PA from the Physical Activity Scale for Persons with Physical Disabilities (25). The scale was adapted slightly by adding explanatory text to clarify the differences between the intensities of PA. In addition, the response format was modified from an ordinal to an interval scale. PA was assessed in terms of days per week and minutes per day and converted into total weekly moderate and vigorous PA minutes. Participants reporting 0 minutes per week on PA were classified as physically inactive.

The influence of *environmental barriers* on life was assessed with fourteen items from the Nottwill Environmental Factors Inventory-Short Form. Response options included: 0 “not applicable” or “no influence”, 1 “made my life a little more difficult”, and 2 “made my life a lot more difficult” (26). Following the procedure described by Reinhardt et al. (27) for assessing environmental barriers across 22 countries using the InSCI survey, the response options were dichotomised into “not applicable/no influence” as 0, and “made my life a little harder/made my life a lot harder” as 1 (27). A sum score was then calculated, ranging from 0 to 14, with higher scores indicating the presence of more environmental barriers.

*Secondary health conditions* (SHCs) were assessed using the Spinal Cord Injury Secondary Conditions scale (28), which was slightly modified in the protocol of the InSCI survey (19). Participants were asked to rate the severity of fourteen conditions: sleep problems, bowel and bladder disorders, urinary tract infections, sexual disorders, contractures, spasticity, pressure ulcers, breathing problems, injuries due to loss of sensation, circulation problems, autonomic dysreflexia, postural hypotension, and pain, on a 5-point scale ranging from 1 “no problem” to 5 “extreme problem”. A sum score was then calculated, ranging from 14 to 70, with higher scores indicating more or more severe SHCs (19).

In addition, participants rated their *general health* in the past 14 days on a 5-point scale, with 1 “Excellent”, 2 “Very good”, 3 “Good”, 4 “Fair”, and 5 “Poor” (19). Responses were dichotomized into “poor” (scores 4–5) and “good” (scores 1–3) to improve interpretability.

*Mental health* and *vitality* were assessed with the Mental Health and Vitality scale of the MOS Short-Form-36, which includes four items on vitality and five on mental health (29). Participants responded to each item using a 5-point scale, ranging from 1 “all of the time” to 5 “none of the time”. Negatively phrased items were reverse coded so that higher scores reflected better mental health and vitality. A total score was calculated and converted into a 0 (“very poor”) to 100 (“excellent”) scale.

*Quality of life* (QoL) was assessed using a single item from the WHOQOL-BREF (30). Participants were asked to rate their QoL in the past 14 days on a 5-point scale from “very poor” to “very good”, with 1 “Very poor”, 2 “Poor”, 3 “Neither poor nor good”, 4 “Good”, and 5 “Very good”. The answers were dichotomised into “poor” (scores 1–3) and “good” (scores 4–5) to improve interpretability.

For all outcomes requiring calculation of a sum score, cases with missing data on any item within the respective questionnaire were excluded from the analysis.

### Statistical analyses

The analyses were conducted using IBM SPSS Statistics, version 28.0 (IBM Corp, Armonk, NY, USA). We used descriptive statistics (mean, standard deviation [SD]; median, interquartile range [IQR], proportions) for demographic characteristics, injury characteristics, and the long-term outcomes per mobility level. Continuous variables were tested for normality using the Shapiro–Wilk test, supported by visual inspection of histograms and Q–Q plots. To evaluate differences in demographic and injury characteristics between mobility levels, we used a parametric test (one-way ANOVA F) for normally distributed data, a non-parametric test (Kruskal–Wallis H) for non-normally distributed data, and the χ² test for dichotomous outcomes. Significance was set at *p* < 0.05 for all analyses.

Differences in long-term outcomes across the mobility levels were analysed using generalised linear models (GLMs). Linear models were applied for interval data (functional independence, participation problems, PA, environmental barriers, SHCs, vitality, and mental health), while binary logistic models were used for dichotomous outcomes (paid work, general health, and QoL). To account for potential confounders, all GLMs were multivariable models, adjusting for age, sex, education level, TSI, and level of injury. These covariates were selected *a priori* based on clinical expertise and supported by evidence from existing literature. Subsequently, *post hoc* pairwise comparisons were conducted to examine specific differences between mobility levels when an overall significant difference was detected. To correct for the number of group comparisons, a Bonferroni correction was applied, adjusting the significance threshold to *p* < 0.0083 (0.05/6).

## RESULTS

Between January and October 2018, 847 individuals were invited to participate in the InSCI-NL survey, of whom 260 responded (30.7%). Mobility level was known in a total of 253 individuals, and those were included in this study. The participants consisted of 42 electric wheelchair users (16.6%), 90 manual wheelchair users (35.6%), 57 ambulatory individuals using walking aids (22.5%), and 64 ambulatory individuals without walking aids (25.3%).

### Demographic and injury characteristics

Table I describes demographic and injury characteristics per mobility level. Mean (SD) age of the total sample was 58.7 (13.6) years, 66.4% were male, and median [IQR] TSI was 10.0 [5.0–22.0] years. Electric wheelchair users were older than manual wheelchair users (*p* = 0.002) and ambulatory individuals using walking aids (*p* < 0.001). There were more men in the manual wheelchair group (77.8%) than in the ambulatory group with walking aids (54.4%; *p* = 0.003). Both wheelchair groups had a longer TSI than both ambulatory groups (*p* < 0.001). The median [IQR] age at injury was 45.0 [28.0–57.0] years and was lower in manual wheelchair users than in both ambulatory groups (*p* < 0.001). A traumatic cause of injury was reported by 62.9% of the participants. The manual wheelchair group consisted of more individuals with traumatic SCI compared with both ambulatory groups (*p* < 0.001 and *p* = 0.002). A complete SCI was present in 28.7% of the participants. There were more individuals with complete SCIs in both wheelchair groups than in the ambulatory groups (*p* < 0.001). Tetraplegia was present in 37.5% of the participants. Electric wheelchair users more often had a tetraplegia than manual wheelchair users (*p* < 0.001) and ambulatory individuals using walking aids (*p* < 0.001).

**Table I:** Demographic and injury characteristics

Factor	*N*	Total sample *n* = 253	Electric wheelchair (1) *n* = 42	Manual wheelchair (2) *n* = 90	Ambulatory using walking aid (3) *n* = 57	Ambulatory without walking aid (4) *n* = 64	*p*-value
Age, years (mean (SD))	253	58.7 (13.6)	66.4 (12.7)	57.4 (13.8)	59.3 (11.7)	54.4 (13.9)	< 0.001^*a^
Sex, male, *n* (%)	253	168 (66.4%)	29 (69.0%)	70 (77.8%)	31 (54.4%)	38 (59.4%)	0.015^*b^
Marital status, married or in partnership, *n* (%)	253	158 (62.5%)	25 (59.5%)	52 (57.8%)	40 (70.2%)	41 (64.1%)	0.473
Education level, higher education, *n* (%)	250	154 (61.6%)	24 (57.1%)	52 (59.8%)	39 (68.4%)	39 (60.9%)	0.657
Age at injury, years (median [IQR])	247	45.0 [28.0–57.0]	46.0 [28.0–63.5]	32.5 [24.0–47.8]	53.5 [39.5–60.8]	47.0 [36.0–56.3]	< 0.001^*c^
Time since injury, years (median [IQR])	247	10.0 [5.0–22.0]	16.0 [6.5–34.0]	20.5 [9.0–30.0]	5.0 [4.0–10.0]	6.5 [3.0–10.3]	< 0.001^*d^
Cause of injury, traumatic, *n* (%)	251	158 (62.9%)	27 (64.3%)	71 (78.9%)	25 (43.9%)	35 (55.6%)	< 0.001^*e^
Completeness of injury, complete, *n* (%)	249	72 (28.7%)	21 (51.2%)	47 (52.2%)	1 (1.8%)	3 (4.8%)	< 0.001^*f^
Level of injury, tetraplegia, *n* (%)	253	95 (37.5%)	27 (64.3%)	23 (25.6%)	17 (29.8%)	28 (43.8%)	< 0.001^*g^

For *post hoc* analyses with Bonferroni correction, statistical significance was set at *p* < 0.0083.

a *Post hoc*: difference between group (1) and (2) (*p* = 0.002), and group (1) and (4) (*p* < 0.001).

b *Post hoc*: difference between group (2) and (3) (*p* = 0.003).

c *Post hoc*: difference between group (2) and (3) (*p* < 0.001), group (2) and (4) (*p* < 0.001).

d *Post hoc*: difference between group (1) and (3) (*p* < 0.001), group (1) and (4) (*p* < 0.001), group (2) and (3) (*p* < 0.001), and group (2) and (4) (*p* < 0.001).

e *Post hoc*: difference between group (2) and (3) (*p* < 0.001), group (2) and (4) (*p* = 0.002).

f *Post hoc*: difference between group (1) and (3) (*p* < 0.001), group (1) and (4) (*p* < 0.001), group (2) and (3) (*p* < 0.001), group (2) and (4) (*p* < 0.001).

g *Post hoc*: difference between group (1) and (2) (*p* < 0.001), group (1) and (3) (*p* < 0.001).

SD: standard deviation; IQR: interquartile range; *N*: total sample included in analyses; *n*: number of participants.

* Significance was set at *p* < 0.05.

### Comparison of long-term outcomes across mobility levels

Table II presents reported long-term outcomes, and the differences between mobility levels adjusted for covariates. A significant difference across mobility levels was found on functional independence in self-care and bladder and bowel management, paid work, participation problems, physical inactivity, environmental barriers, and SHCs. There were no significant differences across mobility levels for time spent on PA, general health, vitality, mental health, and QoL. Of note, the percentage of physical inactivity was high in all subgroups, ranging from 74% in electric wheelchair users to 32% in ambulatory individuals without walking aids. The following paragraphs present comparisons between adjacent mobility levels.

**Table II:** Long-term outcomes per mobility level

Item	*N*	Total	Electric wheelchair (1)	Manual wheelchair (2)	Ambulatory using walking aid (3)	Ambulatory without walking aid (4)	*p*- value^+^
Functional independence: self-care, median [IQR], range 0–20	249	18.0 [12.0–20.0]	6.0 [1.0–10.0]	17.0 [11.5–19.5]	18.0 [17.0–20.0]	20.0 [19.0–20.0]	< 0.001^*a^
Functional independence: bladder and bowel management, median [IQR], range 0–22	221	16.0 [11.0–19.0]	8 [5.0–12.0]	14.0 [9.0–17.0]	19.0 [16.8–20.0]	19.0 [16.5–20.0]	< 0.001^*b^
Paid work, *n* (%), participants < 66 years	167	83 (49.7%)	4 (25.0%)	34 (54.8%)	13 (34.2%)	32 (62.7%)	< 0.001^*c^
Participation problems, median [IQR], range 8–40	216	16.0 [10.0–21.0]	24.0 [17.0–28.3]	15.0 [10.0–19.0]	16.5 [10.0–21.8]	12.0 [9.0–17.5]	< 0.001^*d^
Physical activity: time spent on PA in minutes/week, median [IQR] % Inactive, *n* (%) Of those who are active; time spent on PA in minutes/week, median [IQR]	238 238 135	40.0 [0.0–165.0] 103 (43.3%) 140.0 [60.0–300.0]	0.0 [0–45.0] 28 (73.7%) 162.5 [90.0–270.0]	45.0 [0.0–240.0] 35 (42.2%) 175.0 [62.5–352.5]	60.0 [0.0–165.0] 20 (36.4%) 120.0 [60.0–240.0]	65.0 [0.0–185.0] 20 (32.3%) 145.0 [60.0–300.0]	0.179 0.011^*e^ 0.928
Environmental barriers, median [IQR], range 0–14	240	3.0 [1.0–5.0]	4.0 [3.0–7.0]	3.0 [2.0–5.0]	2.0 [0.0–4.0]	1.0 [0.0–3.0]	< 0.001^*f^
Secondary health conditions, median [IQR], range 14–70	200	26.5 [22.0–33.0]	34.0 [31.0–41.0]	28.0 [23.0–34.0]	27.0 [22.0–31.0]	23.0 [19.0–28.0]	< 0.001^*g^
General health, *n* (%), good to excellent	250	160 (66.4%)	22 (55.0%)	65 (72.2%)	36 (63.2%)	43 (68.3%)	0.390
Vitality, median [IQR], range 0–100	247	62.5 [43.8–68.8]	62.5 [37.5–62.5]	62.5 [50.0–75.0]	56.3 [43.8–68.8]	62.5 [43.8–68.8]	0.937
Mental health, median [IQR], range 0–100	247	75.0 [60.0–85.0]	72.5 [66.3–85.0]	80.0 [60.0–85.0]	75.0 [61.3–85.0]	75.0 [60.0–85.0]	0.386
Quality of life, *n* (%), good to very good	247	170 (68.8%)	21 (52.5%)	66 (76.7%)	37 (64.9%)	46 (71.9%)	0.141

For *post hoc* analyses with Bonferroni correction, statistical significance was set at *p* < 0.0083.

a *Post hoc*: difference between group (1) and (2) (*p* < 0.001), group (1) and (3) (*p* < 0.001), group (1) and (4) (*p* < 0.001), group (2) and (3) (*p* < 0.001), group (2) and (4) (*p* < 0.001).

b *Post hoc*: difference between group (1) and (2) (*p* < 0.001), group (1) and (3) (*p* < 0.001), group (1) and (4) (*p* < 0.001), group (2) and (3) (*p* < 0.001), group (2) and (4) (*p* < 0.001).

c *Post hoc*: difference between group (1) and (4) (*p* < 0.001), group (3) and (4) (*p* < 0.001).

d *Post hoc*: difference between group (1) and (2) (*p* < 0.001), group (1) and (3) (*p* < 0.001), group (1) and (4) (*p* < 0.001).

e *Post hoc*: difference between group (1) and (3) (*p* = 0.009), group (1) and (4) (*p* < 0.001).

f *Post hoc*: difference between group (1) and (3) (*p* < 0.001), group (1) and (4) (*p* < 0.001), group (2) and (4) (*p* < 0.001).

g *Post hoc*: difference between group (1) and (2) (*p* = 0.002), group (1) and (3) (*p* < 0.001), group (1) and (4) (*p* < 0.001), group (2) and (4) (*p* < 0.001).

SD: standard deviation; IQR: interquartile range; *N*: total sample included in analyses; *n*: number of participants.

+ Generalised linear models adjusted for age, sex, education level, time since injury, and level of injury.

* Significance was set at *p* < 0.05.

### Electric wheelchair users vs manual wheelchair users

Adjusted for covariates, independence in self-care, and bladder and bowel management were lower in electric wheelchair users compared with manual wheelchair users (both *p* < 0.001). Furthermore, electric wheelchair users experienced more or more severe participation problems and SHCs (both *p* < 0.001). There were no significant differences between electric and manual wheelchair users regarding paid work, PA, environmental barriers, general health, vitality, mental health, and QoL.

### Manual wheelchair users vs ambulatory individuals using walking aids

Adjusted for covariates, independence in self-care and bladder and bowel management was lower in manual wheelchair users compared with ambulatory individuals using walking aids (both *p* < 0.001). No significant differences were found between the groups for the other long-term outcomes.

Of note, manual wheelchair users reported significantly worse outcomes compared with ambulatory individuals without walking aids on functional independence in self-care and bowel and bladder management (both *p* < 0.001), environmental barriers (*p* < 0.001), and SHCs (*p* < 0.001).

### Ambulatory individuals using walking aids vs individuals without walking aids

Adjusted for covariates, individuals without walking aids were more often engaged in paid work than those using walking aids (*p* < 0.001). No significant differences were found between the groups on the other long-term outcomes.

## DISCUSSION

This study evaluated long-term outcomes in community-living individuals with SCI across different mobility levels in the Netherlands. Our hypothesis that groups with a more independent mobility level would report better long-term outcomes was only partially confirmed. Both functional independence in self-care and bladder and bowel management were, as expected, better for groups with a more independent mobility level, except regarding this comparison between the ambulatory groups. Unexpectedly, other long-term outcomes were not consistently better in groups with more independent mobility levels. Nonetheless, electric wheelchair users generally reported the least favourable outcomes. Most remarkably, ambulatory individuals using walking aids did not report better outcomes than manual wheelchair users, other than for functional independence. Further, no differences between the subgroups were found for time spent on PA, general health, vitality, mental health, and QoL. This latter finding aligns with previous studies suggesting that independent functioning or moving around does not necessarily translate to better outcomes in terms of well-being (5, 31).

In line with previous literature (4, 32) and our hypothesis, electric wheelchair users reported worse long-term outcomes regarding functional independence, participation problems, and SHCs, compared with manual wheelchair users. Although the proportion of electric wheelchair users with paid work was lower than that of manual wheelchair users (25.0% vs 54.8%), this difference was not significant after Bonferroni correction (*p* = 0.012), likely due to the small number of participants below retirement age in the electric wheelchair group (*n* = 16). In contrast to what we expected, ambulatory individuals without walking aids did not report better long-term outcomes than individuals using walking aids, except for paid work. Furthermore, ambulatory individuals using walking aids did not report better long-term outcomes than manual wheelchair users, except for functional independence. Notably, 54.8% of the manual wheelchair users reported having paid work, while this was 34.2% of the ambulatory individuals using walking aids (*p* = 0.056). Despite this difference not being significant when adjusted for covariates, which included age and TSI, the relatively high age at injury and short TSI may still play a role in the relatively low employment in the ambulatory group.

Although mobility level appears to independently influence part of the outcomes, the mechanisms underlying the observed differences remain unclear based on our study. For example, individuals using walking aids may not only have relatively poor walking ability as their main mode of mobility, but may also experience disadvantages related to walking aids (15, 16), and dependence on others for moving around (14), which are not captured in the m-SCIM-SR. Indeed, several other studies reported unique challenges for ambulatory individuals, with and without using walking aids, which may play a role in the reported long-term outcomes. First, ambulating may be strenuous, unstable, and painful, and the quality may be unpredictable (33). Second, individuals with only mild disabilities may have the tendency to mirror individuals without disabilities, which could negatively affect coping and adaptation and could lead to a more negative appraisal of their situation (34). Third, the presence of health issues that are not visible to others may lead to less recognition of the issues, which may negatively affect long-term outcomes (5). Finally, the desire to improve ambulation could potentially contribute to less attention and support to engage in other important aspects of life, such as social and labour integration (6). Notably, Abou et al. (31) reported that manual wheelchair users often report higher satisfaction with social roles and activities than ambulatory individuals with SCI. Their findings suggest that promoting wheelchair use as an alternative mobility mode for individuals with very restricted walking function may support participation and enhance QoL, particularly through greater satisfaction with social roles and activities (31).

Another notable finding was the high prevalence of physical inactivity in all subgroups. Furthermore, at group level, none of the subgroups adhered to the World Health Organization (WHO) PA guideline for adults living with a disability, which recommends at least 150–300 min of moderate intensity or 75–150 min of vigorous intensity aerobic PA per week (35). This confirms previous studies showing low PA levels in both wheelchair users and ambulatory individuals with SCI (36). Interestingly, among those classified as physically active, the wheelchair groups adhered to the WHO guideline, whereas the ambulatory groups did not. This finding should, however, be interpreted with caution, as the physically active wheelchair groups were relatively small (11 electric wheelchair users and 52 manual wheelchair users). Overall, while a smaller proportion of wheelchair users was physically active compared with ambulatory individuals, those who were active tended to engage in more PA.

### Study limitations

The study has some limitations. First, although the response rate (30.7%) and number of participants (*n* = 253) were fair, the subgroups analysed were relatively small. As a result, power may have been insufficient to detect significant differences between groups. Although the Bonferroni correction controls the risk of Type I error when conducting multiple comparisons, it also substantially reduces statistical power by lowering the alpha threshold for each test. Given the relatively small subgroup sizes in our study, this adjustment may have further limited our ability to detect potential differences between groups. However, the selected subgroups provided relevant and new information on long-term outcomes, especially for the ambulatory group. Second, mobility level was operationalised in our study by the ability to move around over a distance of 10–100 m based on the SCIM-SR and divided into four subgroups. This measure of capacity does not provide information on actual functioning in daily life. Especially in the ambulatory group using walking aids, there may have been a large variation in how individuals moved around in their daily lives. Moreover, the survey did not assess the degree of effort required for ambulation, which is a potential determinant of some outcomes in this group. Third, we slightly modified the scoring categories of some of the questionnaires used, in accordance with the protocol of the InSCI survey (20), and we dichotomised some outcomes for better interpretation, which could have affected validity. Finally, self-reported outcomes are inherently subjective, and their reporting may differ between individuals with more dependent and more independent mobility levels. For example, the latter group may rate their outcomes lower as a result of mirroring with individuals without disability and the invisibility of the impairment (34, 37).

### Implications

In the coming years, there will be a growing number of individuals with SCI who can achieve more independent and ambulatory mobility because of advancements in treatment and the increasing incidence of incomplete SCI (38). Although ambulation is a highly valued goal among individuals with SCI, the lack of better long-term outcomes for ambulatory individuals, especially with walking aids, vs manual wheelchair users, points at a need for greater attention to improving long-term outcomes in this ambulatory group. Treatment should be personalised and may include addressing less visible impairments, optimising the selection and fitting of assistive devices, facilitating labour market integration, and supporting emotional adaptation.

Concerning assistive devices, careful selection is essential. For those with very limited walking ability, a wheelchair, primary or supplementary, may provide greater independence, higher levels of PA, and participation. In such cases, training in wheelchair skills should be considered to maximise mobility independence. However, determining the optimal timing of introducing a wheelchair as a (primary) mobility option for those ambulating with difficulty requires careful consideration, as acceptance is unlikely in early rehabilitation but may be appropriate when revisited at longer TSI. Further, labour market integration, an important determinant of QoL (39), could be improved for electric wheelchair users. Finally, to enhance healthy ageing and QoL, PA levels could be improved in all subgroups (40, 41).

### Conclusion

Electric wheelchair users reported overall the least favourable long-term outcomes. Remarkably, while ambulation is a high priority for recovery among individuals with SCI, our results did not show better long-term outcomes for the ambulatory group using walking aids, i.e., individuals with limited walking ability, compared with manual wheelchair users, other than for functional independence. These findings highlight the importance of recognising that ambulation does not necessarily translate into more favourable long-term outcomes, suggesting that rehabilitation teams should carefully consider how to support individuals with walking ability. Future research is needed to examine which specific factors contribute to these outcomes and how to facilitate tailored interventions for ambulatory individuals with SCI.

## References

[1] Bickenbach J, Officer A, Shakespeare T, von Groote P, World Health Organization. International perspectives of spinal cord injury. Geneva: World Health Organization; 2013.

[2] Osterthun R, Post MW, van Asbeck FW, Dutch-Flemish Spinal Cord S. Characteristics, length of stay and functional outcome of patients with spinal cord injury in Dutch and Flemish rehabilitation centres. Spinal Cord 2009; 47: 339–344. 10.1038/sc.2008.127 19002154

[3] Catz A, Itzkovich M, Agranov E, Ring H, Tamir A. The spinal cord independence measure (SCIM): sensitivity to functional changes in subgroups of spinal cord lesion patients. Spinal Cord 2001; 39: 97–100. 10.1038/sj.sc.3101118 11402366

[4] Abou L, Rice LA. The differences in demographics, fear of falling, transfer quality and participation enfranchisement between manual and power wheelchair users with multiple sclerosis and spinal cord injury. Disabil Rehabil Assist Technol 2022; 19 1003–1008. 10.1080/17483107.2022.2138998 36301722

[5] Kifley A, Geraghty TJ, Arora M, Bourke J, Craig A, Cameron ID, et al. Complex lived experiences and hidden disability after spinal cord injury: a latent profile analysis of the Australian arm of the International Spinal Cord Injury (Aus-InSCI) Community Survey. Disabil Rehabil 2023; 6: 4675–4696. 10.1080/09638288.2023.2283101 38018422

[6] Ditunno PL, Patrick M, Stineman M, Ditunno JF. Who wants to walk? Preferences for recovery after SCI: a longitudinal and cross-sectional study. Spinal Cord 2008; 46: 500–506. 10.1038/sj.sc.3102172 18209742

[7] Hardin EC, Kobetic R, Triolo RJ. Ambulation and spinal cord injury. Phys Med Rehabil Clin N Am 2013; 24: 355–370. 10.4103/0972-2327.150605 23598268

[8] Hicks AL, Ginis KA. Treadmill training after spinal cord injury: it’s not just about the walking. J Rehabil Res Dev 2008; 45: 241–248. 10.1682/jrrd.2007.02.0022 18566942

[9] Hicks AL. Locomotor training in people with spinal cord injury: is this exercise? Spinal Cord 2021; 59: 9–16. 10.1682/jrrd.2007.02.0022 32581307

[10] Lawrason S, Tomasone J, Olsen K, Martin Ginis K. ‘I’m glad I can walk, but sometimes it’s so challenging that it’s an inconvenience to myself and others’: physical activity experiences among individuals with spinal cord injury who ambulate. Qual Res Sport Exerc Health 2022; 14: 987–1004. 10.1080/2159676X.2022.2046630

[11] Ames H, Wilson C, Barnett SD, Njoh E, Ottomanelli L. Does functional motor incomplete (AIS D) spinal cord injury confer unanticipated challenges? Rehabil Psychol 2017; 62: 401–406. 10.1037/rep0000146 28581321

[12] Jeawon M, Hase B, Miller S, Eng J, Bundon A, Chaudhury H, et al. Exploring the quality of life of people with incomplete spinal cord injury who can ambulate. Disabil Rehabil 2023; 3: 455–476. 10.1080/09638288.2023.2171495 36740758

[13] Freixes O, Rivas ME, Agrati PE, Bochkezanian V, Waldman SV, Olmos LE. Fatigue level in spinal cord injury AIS D community ambulatory subjects. Spinal Cord 2012; 50: 422–425. 10.1038/sc.2011.175 22270195

[14] Krause J, Carter RE, Brotherton S. Association of mode of locomotion and independence in locomotion with long-term outcomes after spinal cord injury. J Spinal Cord Med 2009; 32: 237–248. 10.1080/10790268.2009.11760778 19810625 PMC2718818

[15] Bateni H, Maki BE. Assistive devices for balance and mobility: benefits, demands, and adverse consequences. Arch Phys Med Rehabil 2005; 86: 134–145. 10.1016/j.apmr.2004.04.023 15641004

[16] Saunders LL, Krause JS, DiPiro ND, Kraft S, Brotherton S. Ambulation and complications related to assistive devices after spinal cord injury. J Spinal Cord Med 2013; 36: 652–659. 10.1179/2045772312y.0000000082 24090470 PMC3831327

[17] Lima R, Monteiro A, Salgado AJ, Monteiro S, Silva NA. Pathophysiology and therapeutic approaches for spinal cord injury. Int J Mol Sci 2022; 23. 10.3390/ijms232213833 36430308 PMC9698625

[18] Devivo MJ. Epidemiology of traumatic spinal cord injury: trends and future implications. Spinal Cord 2012; 50: 365–372. 10.1038/sc.2011.178 22270188

[19] Fekete C, Post MW, Bickenbach J, Middleton J, Prodinger B, Selb M, et al. A structured approach to capture the lived experience of spinal cord injury: data model and questionnaire of the International Spinal Cord Injury Community Survey. Am J Phys Med Rehabil 2017; 96: S5–S16. 10.1097/phm.0000000000000622 28059874

[20] Gross-Hemmi MH, Post MW, Ehrmann C, Fekete C, Hasnan N, Middleton JW, et al. Study protocol of the International Spinal Cord Injury (InSCI) Community Survey. Am J Phys Med Rehabil 2017; 96: S23–S34. 10.1097/phm.0000000000000647 28059876

[21] Cieza A, Kirchberger I, Biering-Sørensen F, Baumberger M, Charlifue S, Post MW, et al. ICF Core Sets for individuals with spinal cord injury in the long-term context. Spinal Cord 2010; 48: 305–312. 10.1038/sc.2009.183 20065984

[22] Osterthun R, Postma K, van den Berg RHJ, Stolwijk J, Tepper M, Post MWM. [Associations between mobility level and levels of health, functioning and well-being]. Ned Tijdschr Revalidatiegeneeskd 2021; 43: 32–36. [in Dutch]

[23] Post MW, Nooijen CF, Postma K, Dekkers J, Penninx F, van den Berg-Emons RJ, et al. People with spinal cord injury in the Netherlands. Am J Phys Med Rehabil 2017; 96: S93–S95. 10.1097/phm.0000000000000619 28059889

[24] Fekete C, Eriks-Hoogland I, Baumberger M, Catz A, Itzkovich M, Lüthi H, et al. Development and validation of a self-report version of the Spinal Cord Independence Measure (SCIM III). Spinal Cord 2013; 51: 40–47. 10.1038/sc.2012.87 22890418

[25] Washburn RA, Zhu W, McAuley E, Frogley M, Figoni SF. The physical activity scale for individuals with physical disabilities: development and evaluation. Arch Phys Med Rehabil 2002; 83: 193–200. 10.1053/apmr.2002.27467 11833022

[26] Ballert CS, Post MW, Brinkhof MW, Reinhardt JD. Psychometric properties of the Nottwil Environmental Factors Inventory Short Form. Arch Phys Med Rehabil 2015; 96: 233–240. 10.1016/j.apmr.2014.09.004. 25264112

[27] Reinhardt JD, Middleton J, Bokel A, Kovindha A, Kyriakides A, Hajjioui A, et al. Environmental barriers experienced by people with spinal cord injury across 22 countries: results from a cross-sectional survey. Arch Phys Med Rehabil 2020; 101: 2144–2156. 10.1016/j.apmr.2020.04.027 32502565

[28] Reinhardt JD, Ballert C, Brinkhof MWG, Post MWM. Perceived impact of environmental barriers on participation among people living with spinal cord injury in Switzerland. J Rehabil Med 2015; 48: 210–218. 10.2340/16501977-2048 26926923

[29] Kalpakjian CZ, Scelza WM, Forchheimer MB, Toussaint LL. Preliminary reliability and validity of a spinal cord injury secondary conditions scale. J Spinal Cord Med 2007; 30: 131–139. 10.1080/10790268.2007.11753924 17591225 PMC2031942

[30] Aaronson NK, Muller M, Cohen PD, Essink-Bot ML, Fekkes M, Sanderman R, et al. Translation, validation, and norming of the Dutch language version of the SF-36 Health Survey in community and chronic disease populations. J Clin Epidemiol 1998; 51: 1055–1068. 10.1016/s0895-4356(98)00097-3 9817123

[31] Development of the World Health Organization WHOQOL-BREF quality of life assessment. The WHOQOL Group. Psychol Med 1998; 28: 551–558. 10.1017/s0033291798006667 9626712

[32] Abou L, Martinez-Navarro O, Kratz A. Satisfaction with social roles and activities across mobility status among persons with spinal cord injury. Spinal Cord 2024; 62: 264–269. 10.1038/s41393-024-00984-9 38519562

[33] Hastings J, Robins H, Griffiths Y, Hamilton C. The differences in self-esteem, function, and participation between adults with low cervical motor tetraplegia who use power or manual wheelchairs. Arch Phys Med Rehabil 2011; 92: 1785–1788. 10.1016/j.apmr.2011.03.023 21762872

[34] Wirz M, van Hedel HJA. Balance, gait, and falls in spinal cord injury. In: Seil FJ, editor. Handbook of clinical neurology. Vol. 159. Amsterdam: Elsevier; 2018. p. 367–384. 10.1016/b978-0-444-63916-5.00024-0 30482328

[35] Buunk AP, Rosario Z, González P. Social comparison, coping and depression in people with spinal cord injury. Psychology Health 2006; 21: 791–807. 10.1080/14768320500444117

[36] Bull FC, Al-Ansari SS, Biddle S, Borodulin K, Buman MP, Cardon G, et al. World Health Organization 2020 guidelines on physical activity and sedentary behaviour. Br J Sports Med 2020; 54: 1451–1462. 10.1136/bjsports-2020-102955 33239350 PMC7719906

[37] Martin Ginis KA, Papathomas A, Perrier M-J, Brett S, Smith B, SHAPE-SCI Research Group. Psychosocial factors associated with physical activity in ambulatory and manual wheelchair users with spinal cord injury: a mixed-methods study. Disabil Rehabil 2017; 39: 187–192. 10.3109/09638288.2015.1045991 26104107

[38] Jannings W, Pryor J. The experiences and needs of persons with spinal cord injury who can walk. Disabil Rehabil 2012; 34: 1820–1826. 10.3109/09638288.2012.665126 22423597

[39] Quddusi AI, Wilson JR, Furlan JC, Klurfan P, Deng J, Waheed H, et al. Shifting demographics, characteristics, and outcomes of traumatic spinal cord injury: a systematic review. Spinal Cord 2026; 64: 211–221. 10.1038/s41393-026-01182-5 41731066

[40] Escorpizo R, Naud S, Post MWM, Schwegler U, Engkasan J, Halvorsen A, et al. Relationship between employment and quality of life and self-perceived health in people with spinal cord injury: an international comparative study based on the InSCI Community Survey. Spinal Cord 2024; 62: 110–116. 10.1038/s41393-023-00953-8 38160224

[41] Stevens SL, Caputo JL, Fuller DK, Morgan DW. Physical activity and quality of life in adults with spinal cord injury. J Spinal Cord Med 2008; 31: 373–378. 10.1080/10790268.2008.11760739 18959354 PMC2582427

[42] Pelletier C. Exercise prescription for persons with spinal cord injury: a review of physiological considerations and evidence-based guidelines. Appl Physiol Nutr Metab 2023; 48: 882–895. 10.1139/apnm-2023-0227 37816259

